# Incidents related to safety in mental health facilities in Kenya

**DOI:** 10.1186/s12913-023-09074-7

**Published:** 2023-01-28

**Authors:** Kamaru Edith Kwobah, Sitienei Robert Kiptoo, Florence Jaguga, Felicita Wangechi, Saina Chelagat, Francis Ogaro, WK Aruasa

**Affiliations:** Moi Teaching and Referral Hospital, 3 Eldoret, Eldoret, 30100 Kenya

**Keywords:** Mental health facilities, Safety, Prevention, Mental disorders, Incidents

## Abstract

**Background:**

Both patients and health care providers working in mental health facilities witness high rates of incidents that have the potential to jeopardize their safety. Despite this, there are few studies that have documented the kind of incidents that are experienced, or explored the potential contributors to these incidents, and solutions that would result in better safety. This study explored various types of safety related incidents occurring in mental facilities in Kenya, perceived contributing factors, and recommendations for improve.

**Methods:**

This qualitative descriptive study was carried out between December 2019 – February 2020. It included 28 mental health staff across 14 mental health unit spread across the country.

**Results:**

All the participants reported having personally experienced an incident that threatened their safety or that of the patients. Most of the respondents (24/26. 91.67%) admitted to have experienced verbal aggression while 54.17%, (*n* = 24) had experienced physical assault. Participating health care workers attributed the safety incidents to poor infrastructure, limited human resources, and inadequate medication to calm down agitated patients. Suggested solutions to improve patient safety included; improving surveillance systems, hiring more specialized healthcare workers, and provision of adequate supplies such as short-acting injectable psychotropic.

**Conclusion:**

Incidents that threaten patient and staff safety are common in mental health facilities in Kenya. There is need to strengthen staff capacity and reporting mechanisms, as well as invest in infrastructural improvements, to safeguard patient and staff safety in mental health facilities in Kenya.

## Introduction

Incidents affecting the safety of patients and health care workers within mental health facilities are a major concern [1]. While mental health facilities should not be assumed to be extremely dangerous places, a wide range of incidents have been reported by staff working in mental health facilities. The incidents range from verbal threats, stalking, physical assault, and sexual harassment or rape [2–4]. One study done in South Africa reported the prevalence of violence among long-term inpatients was 16% and that fighting among patients was the most common incident [5]. Additionally, a survey conducted in a Botswana psychiatric hospital reported the rate of physical violence among mental health workers in Botswana was high (69.8%) with the nurses being the most affected members of staff [6]. A study done in New Zealand reported that verbal aggression was experienced by 93% of healthcare workers while physical aggression was experienced by 65% of workers [7]. Many incidents have been reported in the process of restraining aggressive patients hence staff are more frequently the victims [8].

These incidents have the potential to cause both physical and psychological harm to patients and healthcare workers [9, 10]. They may contribute to low morale among staff, contribute to high rates of sick leave and high staff turnover and leave staff with negative feelings such as guilt, self-blame, and insecurity in dealing with the patient or even feeling of failure in their professional work [11]. In addition, aggressive incidents have been reported to have considerable financial implications due to infrastructural damage as well as the cost of treatment of injuries [12].

Existing literature has linked safety incidents to both patient and staff factors. A study conducted in Quebec city reported severity of cognitive impairment of the patient as an important contributor to both physical and verbal aggressive tendencies [13]. Staff factors linked to safety incidents include training of the staff, and duration of employment [6].

The reporting rates of safety incidents in mental health facilities is low, which means there is no strong evidence base to support a case for investing in interventions to promote safety for both patients and staff in mental health facilities [14].

In Kenya there is no study that has been done on safety incidents within mental health facilities.

The current study sought to: Establish the types and frequency of safety incidents occurring in mental health facilities in Kenya, establish perceived factors associated with the occurrence of these incidents and finally to gather health provider opinions on ways of reducing incidents in mental health facilities. This work is important in informing efforts to improve safety and is in line with the Quality Rights Initiative which was recently launched in Kenya that seeks to improve the quality of mental health services in the country [15].

## Methods

### Study design

This was a qualitative study.

### Study site

The study targeted level 4, 5 and 6 hospitals in Kenya that provide mental healthcare services. There are six levels of health care facilities in Kenya managed by both the national and county governments under the devolved systems. The first five levels are managed by the county governments while the sixth level is managed by the national government. In this system, the patients may move from one level to the next by using a referral letter. Level four and five hospitals are the county referral hospitals which are the highest health facilities managed by the county governments. Services offered at this level include provision of training services for health workers, referral services for curative and specialized care functions, management and coordination support to the counties and provision of internship.

#### Respondents

The respondents were key mental health personnel working at the selected facilities including psychiatrists, psychiatric nurses, psychiatric clinical officers, psychologists and or psychological counselors, and occupational therapists, depending on the cadres deployed in the different facilities.

### Eligibility criteria

We included personnel working at the mental health facilities for a period of not less than 12 months by the time of interview and were involved in day to day patient care within the mental health facilities. Staff working in administration only but not involved in direct care of patients were excluded from participating in the study.

### Sample size and sampling

Twenty six key informant interviews were conducted that involved two personnel per facility. The personnel were purposively selected.

### Data collection tool

An interview guide developed by the research team was used to collect data on the staff experiences with safety incidents, perceived contributing factors, and recommendations for improving staff safety. Data was also collected on participant demographics and facility characteristics. The interviews were conducted face to face by a psychiatrist. The interviews were audio-recorded and field notes taken.

### Data management and analysis

Qualitative data was transcribed and coded manually by two coders with experience in qualitative research. All qualitative data were de-identified prior to analysis. Emergent themes were determined and through iterative reading of data, recurrent themes were identified.

## Results

### Characteristics of mental health facilities

Data was collected from 26 staff working in 13 mental health facilities across Kenya as shown in Table1.

**Table 1:** Mental health infrastructure, bed capacity and bed occupancy

Facility Name	Facility Level	Mental Health Infrastructure	Bed capacity	Bed occupancy
Mathari National Teaching & Referral Hospital	Level 6	Stand-alone Mental Hospital	740	100
Moi Teaching & Referral hospital (MTRH)	Level 6	Stand-alone Mental Health Unit with a general hospital	80	100
Kenyatta National Hospital (KNH)	Level 6	Patients hosted within a general ward	No specific beds allocated for mental health patients	could not be determined
Meru	Level 5	Stand-alone Mental Health Unit within a general hospital	17	100
Embu	Level 5	Mental health patients hosted within a general ward	16	80
Machakos	Level 5	Patients hosted within a general ward	22	150
Kisii	Level 5	Stand alone Mental Health Unit within a hospital	21	100
Kisumu	Level 5	Stand alone Mental Health Unit within a hospital	30	100
Kakamega	Level 5	Stand alone Mental Health Unit within a hospital	21	100
Thika	Level 5	Patients hosted within a general ward	0	0
Nakuru	Level 5	Stand-alone Mental Health Unit within a hospital	25	230
Nyeri	Level 5	Stand-alone Mental Health Unit within a hospital	24	80
Gilgil	Level 4	Stand-alone Mental Health Unit within a hospital	50	110

The facilities included in the study ranged from level 4 (Sub County Hospital) to level 6 (Teaching and referral hospitals). The services offered in these facilities included outpatient and inpatient services as well as follow-up clinics for patients admitted and discharged.

There were a total of 359 health care providers in the 13 facilities assessed. Majority of them were nurses (56%, *n* = 359). The distribution of the health care providers differed between the facilities with nurses being present in all the facilities. Most of the facilities had a psychiatrist 92.3%, 1(2/13). Approximately 60% had a psychologist whereas 76.9% had a social worker while more than half (53.9%, *n* = 13) had clinical officers assigned to the unit. Additionally, majority of the facilities (84.6%, 11) had access to the services of an occupational therapist and 69.2% (*n* = 13) had security officers although 23.1% of these relied on security officers serving the entire hospital. 30.8% [4] of the facilities did not have security officers.

### Incidents in mental health facilities

Most of the respondents (24/26. 91.7%) admitted to have experienced verbal aggression while 54.2%, (*n* = 24) had experienced physical assault. Most of the facilities reported that they have had patients absconding (88.5%, *n* = 13). The frequency of occurrence differed between the facilities depending on the structure and organization of the facilities and therefore did not exhibit a particular pattern. The frequency ranged all the way from rare to frequent with 29.2% (*n* = 13) of the facilities having had more than 10 absconding patients in a year. Most of the facilities reported that accidental falls had either never happened at their facilities (37.5%, *n* = 13) or happen under rare circumstances (41.7%, *n* = 13). A third of the facilities (39.1%, *n* = 13) reported incidents of patients dying of unknown causes but this was reported that it occurs under rare circumstances. Most of the facilities (58.3%, *n* = 13) had experienced a patient that had tried to harm themselves deliberately with 41.7% of these being reported as rare (occurring between 1–4 times in a year. Most of the facilities reported that they have had patients absconding (88.5%, *n* = 13).

The emerging themes in this study were identified as shown in Table 2 below giving a summary of the various types of incidents reported, factors contributing to the mental health incidents and staff recommendation for improving safety.

**Table 2:** Types of incidents, contributing factors and recommendation for improving safety

Type of incidents	Incidents directed to staff by patients	Physical assaults Sexual assaults Verbal assaults
	Incidents between patients	Physical assaults Sexual assaults Verbal assaults
	Incidents directed to patient by staff	Physical assaults Sexual assaults Verbal assaults
	Other incidents	Absconding Sudden death Suicide Deliberate self harm Accidental fall
Contributing factors	Patient factors	Prolonged stay, alcohol and substance abuse, delays in receiving care, unstable mental state, patient’s own biases
	Staff factors	Shortage of staff, inadequate security mechanism, stigma for mental illness
	System factors	Deploying staff with challenges, lack of support from management, medication stock outs
	Infrastructural factors	Poorly designed facilities, lack of seclusion rooms, inadequate working space, inadequate bed capacity, close proximity of male and female wards
Recommendation for improving safety	Improving infrastructure Addressing staff shortage Employment of staff with specialization Availability of adequate supplies Psycho-education Adequate funding and direct budgetary allocation	

Respondents reported to have experienced various types of incidents involving staff and patients ranging from physical, sexual and verbal aggression as quoted below.

### Incidents directed to staff by patients

Incidents directed to staff by patients involved physical, sexual and verbal aggression.

#### Physical aggression

“… There was a patient who was very aggressive. We had tried medication and they were not responding… he slapped the two nurses and they ran away…” (Male Respondent, Nyeri).

#### Sexual aggression

“There's a patient that locked the door to one of the dorm rooms and said that he wanted to sleep with the nurse that day. She lied to him that she would go get ready and then come back. He allowed her to leave. She was traumatized and she had to get psychological help “ (Female Respondent, Gilgil).

#### Verbal aggression

“… we were giving medication with one of my colleagues when one of the patients declined to take the medicine and started attacking my female colleague saying that she doesn’t want to be served by a female nurse. She started saying this unwritten words to my colleague…”( Male respondent, MTRH).

### Incidents between patients

Incidents reported between patients also ranged from physical, sexual and verbal aggression.

#### Physical aggression

“…this was an alcoholic patient and he developed hallucinations and then he started attacking other patients, he was going punching other patients in the wards and throwing away their linen…” (Female respondent, MTRH)

#### Sexual aggression

“There were two patients of about 20, I would say they were in their early 20 s, they befriended each other and this day they were found romancing each other, so we are not sure if they had had sex already” (Male Respondent, Embu)

#### Verbal aggression

“Verbal aggression happens almost daily between patients. Especially the manic patient, they can get very aggressive” (Female respondent, Gilgil).

### Incidents directed to patient by staff

Respondents reported having had experiences of staff displaying aggressive behavior towards patients which ranged from physical, sexual and verbal aggression.

Concerning physical aggression, one respondent narrated a particular incident involving self.

“Yes, and it was some kind of a punishment. Like for me this morning when the patient took my glasses and threw the glasses in the floor, so I was provoked and I felt that I should punish the patient. I took the patient in the strong room and gave him some beating. He is a student and I told him not to repeat such a thing again” (female respondent, Kakamega)

Sexual aggression was reported to be rare but has ever happened within the facilities.

“Sexual aggression by staff towards patients is rare. I have only witnessed it once in my career” (Female respondent, Kakamega)

#### Verbal aggression

“This happens sometimes. Every so often, a staff may be heard using negative language when provoked by the patients’ behaviour” (Male respondent, Kisumu)

### Other incidents

Incidents of deliberate self harm were reported in some of the facilities.

“We had an issue just a few weeks ago of a patient who was taking cocaine and you know very well the side effects of cocaine. So, this patient was trying to cut himself on the neck” (male respondent, Kisumu)

Attempted suicide was also reported within the facilities.

“…She had asked for a blade to cut her nails but she used it to slit her throat and she succumbed to her injuries later…” (Female Respondent, Nakuru)

Incidents of patients absconding was a common occurrence reported by some of the participants.

“…it was common for patients to abscond, especially the tall ones, they could make a ladder using other patients or use the window. There have been modifications to the perimeter wall and now this has reduced. However some try to walk out through the main door…” (Female respondent, MTRH)

Accidental falls was also reported in some of the facilities.

“What can also make them fall is medication. You realize how the drugs work, they make people drowsy. The patient might decide to wake up, but still very drowsy. In the process they fall” (Female respondent, Nakuru)

Sudden death had been witnessed in the mental health facilities

“I was informed that the patient previously had been aggressive. The team had secluded her due to aggressive behaviour. They had given her medication and she had even eaten lunch. So, when they were going to give her the evening medication, they found that she had passed away. Her vitals and been taken that day they were normal …” (female respondent, Gilgil)

### Factors contributing to the mental health incidents

Participants attributed incidents in mental units to various patient, staff, system and infrastructural factors.

#### Patient related factors

Extended hospital stay was reported in several facilities and this was a concern especially when discharge process was delayed.

“… some patients have been discharged and they are not taken home immediately so a patient may feel like they have been neglected. Some of them end up jumping over the wall and absconds…”(Female respondent, Kakamega)

A diagnosis of Alcohol and drug abuse was mentioned by respondents in all the facilities.

“… patients with drug abuse come tell us this medicine we are giving them is of no use, because when they go back to university, they are still going to take the drugs they were taking. It is really difficult to handle patients with drug abuse…”(Female respondent, Kisii)

Perceived delays in receiving care by patients especially concerning reviews and diagnostic investigations were reported in some of the facilities.

“…The long waiting time is one possible contributor. They feel they are being delayed…you know for one psychiatric patient you can take so many hours to assess and others end up being aggressive …” (Female Respondent, KNH)

Unstable mental state of the patient was mentioned by some of the respondents to contribute to the aggressive incidents.

“…There is one who confused me with the wife with whom they had separated and so he directed his anger to me, and started calling me a prostitute…” (Female respondent, MTRH)

Patients own biases in some circumstances may lead to aggression.

“… we were giving medication with one of my colleagues and one of the patients declined to take the medicine and attacked my female colleague saying that she doesn’t want to be served by a female nurse and she started saying using vulgar words to the colleague…”( Female respondent,MTRH)

#### Staff Related factors

Shortage of staff was a major concern reported by respondents in most of the facilities.

“… we don’t have enough mental health nurses in the ward. We are only two. The others are just normal staff working on on-job training…” (Female respondent, Kakamega)

Inadequate security mechanisms was also mentioned occasioning lapses in the process of care thus contributing to incidents of patient absconding and committing suicide.

“…we only have one security guard at the gate and sometimes they are not there, so the patients find an easy way out of escaping through the gate…”(Male Respondent, Meru)

“… There is a time a patient came and a search was not done. She had some medicines she had come with from home. She still had about 10 tablets which she swallowed them all…”(Male respondent, Kisumu)

Stigma for mental illness especially among staff hence very few are willing to specialize or work in mental health units.

“…Stigma is the major one, such that there are no staff willing to be deployed at the mental unit and if someone is forced then they end up mishandling the patients…”(Male respondent, Kisumu)

#### System related factors

Deploying staff with challenges including physical disabilities or those who have advanced in age and are almost retiring or on contract post retirement may not have the energy to handle some of the psychiatric emergencies efficiently.

“… the challenge is when you have nurses that are nearly retiring and the ones that are physically disabled. We are not trying to discriminate people with physical disabilities but psychiatry needs people who are physically fit…We have some that have retired and have been added five extra years…”(Female respondent, Kisii)

Lack of support from management was also raised by some of the respondents.

**“…** have everyone on board. Listen to our problems because we have our own issues. it’s a tiring exercise, we need to vent, listening to problems… it’s not an easy thing but we try…” (Female Respondent,Thika).

Medication stock outs making it difficult to effectively manage patient symptoms were reported in some of the facilities.

“… I can say lack of medication which helps them calm down faster…you can find them getting aggressive when the medications are out of stock leading to more incidents…”(Female respondent, Gilgil)

#### Infrastructural related factors

Poorly designed facilities were reported to contribute to some of the incidents including patients absconding in some of the facilities.

“…some have insight and so they look for ways of going out. some sneak out… they go up the security wall…” (Female Respondent, Nyeri)

Lack of seclusion rooms in some of the facilities making it difficult to restrain or seclude physically aggressive patients to calm them down.

“…other factors like lack of enough strong rooms…the patients that come and we only have two that are already in the strong rooms. if we receive more, then we are not able to lock them up…”(Female Respondent, Meru)

Inadequate working space was reported to limit the staff in providing healthcare services efficiently. One particular respondent narrated the challenges of operating in one room.

“…We do not even have safe rooms… this is the only room that serves as an injection room and we do everything here. If we have counselling we do it here, so space is a challenge…” (Male Respondent, Thika)

Close proximity of male and female wards in some of the facilities was reported to contribute to some incidents of sexual aggression between patients.

“…the female and the male wards are almost in the same place although we have different sleeping places, but they are so close it almost looks like it is the same place which increases chances of patient to patient incidents…”(Male Respondent, Embu)

Inadequate bed capacity was also mentioned by most of the respondents to contribute to aggressive incidents.

“… patients were sharing beds. You could find one patient fighting another because of the sharing. Maybe the patient was hallucinating and decided to beat the other…” (Female Respondent, Kakamega)

“… I would say it is because of the congestion in the wards…our bed capacity is small and so others have to sleep on the floor. It is usually the main complain…” (Male Respondent, Nakuru)

### Staff Recommendations for improving safety

Staff proposed that improving infrastructure, addressing staff shortage and adequate support from management would reduce the incidents in mental facilities.

Improving infrastructure to enhance efficiency in provision of care addressing patient needs as they arise was mentioned by all the respondents.

“…, We can consider having a CCTV within the unit, this will help the nurse manager at the station to run the wards easily. She is able to monitor patients at every corner of this facility…”(Male Respondent, Embu)

#### Address staff shortage

“…We need to increase the number of staff. For example there should be a 24-hour gateman at the unit gate… we need to increase the number of security personnel in the unit… we need to increase the number of staff so that at each and every particular shift according to me, there should be at least three qualified staffs…”(Male Respondent, Kisumu)

Employment of staff with specialization in mental health equipped with knowledge and skills in managing psychiatric patients to ensure efficiency in provision of care was mentioned by most of the respondents.

“… Teaching institutions should make it mandatory so that all staff get some training on mental health… People should also go for specialization…”(Female Respondent, Machakos)

Ensuring availability of adequate supplies will help in handling patient symptoms effectively.

“…We need drugs to be there all through…sometimes we are still using the old generation drugs. We don’t have the new generation drugs…even the few that we have are only brought for some-time and then they run out of stock…” (Female Respondent, Kakamega)

Psycho-education on the nature of mental illness and management was mentioned by some of the participants.

“… Educating the ones who are there because they are not enemies, just to understand that their safety is in their hands and they must be careful…” (Male Respondent, Nakuru)

#### Adequate funding and direct budgetary allocation

“…Maybe the government can think of subsidizing hospital fee for the mental health cases or for those who are not able to engage in any gainful employment because of their mental conditions, then they can be considered under the disability welfare system…” (Male Respondent, Embu)

## Discussion

To the best of our knowledge, this is the first study that has documented safety incidents in mental health facilities in Kenya. The findings provide evidence that safety incidents are frequently experienced within mental health facilities, and these experiences can be attributed to various staff, patient and system factors. The study also provides suggestions by the healthcare providers that can be implemented to improve safety in these facilities.

### Types and frequencies of incidents

Our study established that incidents that threaten the wellbeing of both staff and patients are common in mental facilities in Kenya. A recent systematic review that included 146 studies reported a wide range of between 8–76% [16]. A study done in Italy reported that one out of ten workers in mental health facilities had experienced physical aggression from patients, and one out of three were exposed to non-physical violence in the previous year [17]. In a study done in Switzerland among 2017 patients a total of 760 aggressive incidents were reported [18].

In our study the most reported incidents were verbal aggression followed by physical and sexual related incidents. This is similar to a study done in West Africa that found that about half of mental health care providers had experienced physical assault by patients at least once within their employment period in the psychiatric facility, and 33.7% had been physically assaulted in the past 12 months pointing the inherent risk of aggression in psychiatric units [19]. A study done in Massachusetts reported that 85.3% of the reported incidents experienced by healthcare workers were physical assaults,1.2% were sexual aggression, 1.7% were nonverbal intimidation and 6.0% were verbal aggression [20]. These incidences are worrying as they have negative consequences on the mental health of both patients and staff [21].

Our study found that most of the facilities have had patients absconding from the mental facilities. A study done in South Africa reported an absconding rate of 7.6 of the admitted patient [22]. Similarly, a multicenter study done in Australia found that incidents of absconding were 15.7% of all the admissions and these episodes peaked in the second week following admission and was associated with drug and alcohol disorder, younger age, and longer periods of hospitalization [23]. Absconding is an area of concern as it may translate to higher risk of self-harm, violence, non-adherence, relapses, substance use and negative media attention for the affected facilities [24].

In more than half of the facilities in our study at least one patient had tried to harm themselves, attempted suicide and complete suicide. This is consistent with findings of a previous meta-analysis which reported that inpatient suicides are common [25, 26]. A case control study done in Austria reported a suicide rate of 45.7 per 100,000 admissions [27]. These incidences have been linked to the mental illness of the patient and are believed to cause distress to staff that mental health facilities are expected to be safe spaces [28].

Facilities in our study reported incidences of sudden deaths. In a 23 year retrospective study in Egypt, sudden unknown causes of death were reported in 41.3% of the assessed records [29]. In a retrospective structured root cause analysis done in a large psychiatric hospital in New York, it was found that incidents of sudden and unexpected deaths among psychiatric patients had increased greatly especially among patients with co-morbid medical conditions [30], and warrant well powered longitudinal studies to understand the circumstances better.

In our study, accidental falls were also reported in a number of facilities. A study done in Thailand reported that falls incidents were more frequent at night and within the first week of admission and that common activities during falls were bathroom-related and getting up from the bed or chair [31]. A retrospective review of Charts in Belgium reported 4.4 falls per 1,000 patient days, and these falls were linked to mental status of the patients [32].

### Factors associated with incidents

#### Patient factors

Prolonged stay at the hospital after recovery and discharge due to financial constraints was reported as a potential contributor to safety incidents. Some of these patients may have previously used violence when they feel that their needs are not being addressed in their home environment and may continue engaging in aggressive behavior even when hospitalized as they may incorporate the care givers in their psychotic symptoms [33]. Prolonged stay in hospital may increase agitation due to the restrictive nature of the mental health facilities [34].

A diagnosis of alcohol and substance use was linked to aggressive incidents by some participants. This is in agreement with a systematic review which reported a history of substance use as one contributor to aggressive tendencies [35]. A previous study in Ethiopia reported the prevalence of aggressive behavior among patients with a diagnosis of substance use disorder was 37.9% [36]. Patients with substance use disorder may develop symptoms of withdrawal resulting in aggressive behavior, but they may also have personality traits that contribute to aggressive incidents [37].

Acute phase of illness and unstable mental state of the patients were also reported to contribute to the aggressive incidents. A study done in Ethiopia attributed these aggressive behaviors to the psychopathological symptoms such as delusions, hallucinations and other clinical symptoms affecting their mental state especially in the acute phase of the illness [38].

Mental disorders especially psychotic disorders, mood disorders and personality disorders have been linked to increased risk of deliberate self harm and suicide mental facilities [39].

#### Staff factors

Staff factors such as shortage, lack of training and students doing their clinical attachment were perceived to contribute to incidents of aggression. A previous study found a positive relationship between aggressive incidences and more staff without psychiatric training [40]. Other studies have linked age of the staff and the length of experience with increase likely hood of being involved in an aggression attack [41]. In a ten year analysis of staff victims of patient aggression in psychiatric settings, the findings suggested that junior male staff with less formal education and training were at highest risk [42]. In our study students were more likely to be affected by these aggressive incidents which could be due to their new encounter with mentally sick patients and also spending long contact hours with patients during their shift as they learn [43].

#### System and infrastructural factors

Delay in service provision was also reported as a contributor to incidents. This finding is similar with findings of a study done in a Finish forensic psychiatry hospital that reported that aggressive incidents occur often at specific times of the working shift when patients experience some sense of delays [44]. High number of patients and overcrowding was also seen as a contributing factor in our setting. A study done in China identified overcrowded wards and shortage of psychiatric beds as a contributor to increase in aggressive incidents [45]. A poorly congested facility that prevents privacy, is noisy and has other stressful features can intensify the stress of mental illness and together with involuntary confinement may worsen aggression [46]. A study done in United Kingdom found the association of all types of patient aggression with high patient turnover [47].

### Staff recommendations for improvement of safety in mental health facilities

#### Increasing trained human resource for mental health

Respondents suggested the need to address staff shortage and build capacity through frequent continuous professional education and in-service short trainings as well as provide opportunities and incentives for the staff already working in mental units to be trained on mental health. This may go a long way in improving the quality of care and thus address staff related factors contributing to incidents of aggression. In a retrospective study analyzing the characteristics of staff victims of psychiatric patient assaults it was found that staff tended to be less experienced and less trained as mental health worker or being trainees of differing disciplines [42]. To promote occupational and patient safety in psychiatric settings, the curriculum for training mental health workers should ensure adequate skills training for screening patients at risk for violence and implementing strategies to reduce aggressive incidents [45].

#### Improved supplies

The respondents emphasized the need to ensure availability of adequate supplies to efficiently address patients’ needs thus enhancing staff and patient safety. Additionally, having unit programs with structured patient activities, reliable routines and consistency in staff attitudes is key in reducing aggressive incidents [48, 49].

#### Improved infrastructure

Suggested improvement on infrastructure included increasing bed capacity, increasing seclusion rooms to safely restrain potentially violent patients and having perimeter wall to avoid absconding. This would improve the ward environment making it more conducive for patient recovery and allow staff to easily monitor patients and provide timely care to mitigate these incidents.

#### Improving security and surveillance systems

Suggestion of hiring more security personnel is key because they play an important role in providing a show of authority that would deter patients from aggressive behavior. In addition, improving surveillance through use of CCTV would allow staff to see through many areas of the building hence allow them to address any safety issue in a timely manner. To the extent possible, institutions would also consider having rapid response teams and flying squad that can be mobilized within a short period in situations of extremely aggressive incidents.

### Limitations

While this study describes for the first time the safety incidents reported in mental facilities in Kenya, it is not without limitations. The data for this study was obtained from a small sample using qualitative methods thus limiting generalizations of the results. Being an observational study cause and attribution for the incidents cannot be discussed. We recommend well powered longitudinal studies to better understand factors contributing safety incidents in mental health facilities, as well as implementation studies to evaluate feasible interventions that can promote patient and staff safety. We also recommend a study analyzing specific characteristics of patient and staff involved in the safety incidents like age, gender, diagnosis, length of hospital stay, prescribed medication, staff experience, level of training, among others in order to better understand causes and contributing factors for aggressive behavior among psychiatric patients. An interventional study exploring mitigation strategies against safety incidents in mental health facilities will help identify effective measures to promote safety and enhance quality health care.

## Conclusion

Findings from this study show that safety incidents in mental health facilities in Kenya are a common phenomenon affecting both patients and staff. Various factors are acknowledged as potentially contributing to the current situation, most of which can be modified. There is need to put in measures to improve the safety, targeting all the contributing factors. Greater investment in mental health to improve systems and infrastructure, capacity building for staff to handle the incidents has the potential to improve safety in mental health facilities.

## Data Availability

The datasets used and/or analyzed during the current study are available from the corresponding author on reasonable request.
